# Lung Ultrasound in the Diagnosis of Pleural Tuberculosis

**DOI:** 10.31662/jmaj.2026-0143

**Published:** 2026-08-07

**Authors:** Chee Yik Chang

**Affiliations:** 1 Infectious Disease Unit, Department of Medicine, Hospital Sultanah Aminah, Johor, Malaysia

**Keywords:** lung ultrasound, pulmonary tuberculosis, pleural tuberculosis

## To the Editor

I read with great interest the article by Ohnishi et al. ^(1)^ describing a case of tuberculous pleurisy detected using lung ultrasound. Pulmonary tuberculosis (PTB) remains a significant global health concern, particularly in endemic regions such as Southeast Asia. While chest radiography and computed tomography remain the standard imaging modalities, lung ultrasound has gained increasing recognition as a valuable bedside tool. Its utility lies in detecting abnormalities that extend to the pleural surface, making it especially useful in critically ill patients and resource-limited settings.

A key ultrasound feature of PTB is subpleural consolidation. These appear as hypoechoic, tissue-like areas disrupting the normally smooth pleural line. They may be small, nodular, or larger with irregular margins. Within these consolidations, air bronchograms can be seen as bright, punctate, or linear echoes, representing air within inflamed bronchi. These findings are typically patchy and asymmetrical, although ultrasound is limited to peripheral lung regions ^(2)^.

Pleural line abnormalities are also common. In PTB, the pleural line may appear thickened, irregular, or fragmented due to adjacent inflammation. This contrasts with the smooth pleural line in normal lungs. B-lines, vertical reverberation artefacts arising from the pleural line, may be present and usually occur in a focal distribution, reflecting localized interstitial involvement rather than diffuse disease ^(3)^.

Pleural effusion is a frequent manifestation and is readily identified on ultrasound. It appears as an anechoic or complex collection in the pleural space. Tuberculous effusions often show internal echoes, septations, and fibrin strands, indicating an exudative process. Ultrasound is also valuable for guiding safe thoracentesis, thereby facilitating the diagnosis of pleural tuberculosis ^(4)^. Diagnostic thoracocentesis is also essential for differentiating malignant pleural effusion from tuberculous effusion ^(5)^.

Lung ultrasound is also useful for treatment monitoring, enabling serial assessment of the resolution of consolidations and pleural effusions. However, it remains operator dependent and is limited in evaluating deeper lung parenchyma. In conclusion, lung ultrasound is a valuable adjunct in the assessment of PTB, particularly in tuberculosis-endemic settings.

## Article Information

### Author Contributions

Chee Yik Chang: conception and design of the study, acquisition of data, drafting the manuscript, and approval of the final version.

### Conflicts of Interest

None
